# Gender Differences in Bile Acids and Microbiota in Relationship with Gender Dissimilarity in Steatosis Induced by Diet and FXR Inactivation

**DOI:** 10.1038/s41598-017-01576-9

**Published:** 2017-05-11

**Authors:** Lili Sheng, Prasant Kumar Jena, Hui-Xin Liu, Karen M. Kalanetra, Frank J. Gonzalez, Samuel W. French, Viswanathan V. Krishnan, David A. Mills, Yu-Jui Yvonne Wan

**Affiliations:** 1 Department of Medical Pathology and Laboratory Medicine, University of California, Davis, Sacramento, CA USA; 2 Department of Food Science and Technology, Department of Viticulture and Enology, University of California, Davis, CA USA; 3 Laboratory of Metabolism, Center for Cancer Research, National Cancer Institute, NIH, Bethesda, MD USA; 4 Department of Pathology, Harbor UCLA Medical Center, Torrance, CA USA; 5 Department of Chemistry, College of Science and Mathematics, Fresno State University, Fresno, CA USA

## Abstract

This study aims to uncover how specific bacteria and bile acids (BAs) contribute to steatosis induced by diet and farnesoid X receptor (FXR) deficiency in both genders. A control diet (CD) and Western diet (WD), which contains high fat and carbohydrate, were used to feed wild type (WT) and FXR knockout (KO) mice followed by phenotyping characterization as well as BA and microbiota profiling. Our data revealed that male WD-fed FXR KO mice had the most severe steatosis and highest hepatic and serum lipids as well as insulin resistance among the eight studied groups. Gender differences in WD-induced steatosis, insulin sensitivity, and predicted microbiota functions were all FXR-dependent. FXR deficiency enriched *Desulfovibrionaceae*, *Deferribacteraceae*, and *Helicobacteraceae*, which were accompanied by increased hepatic taurine-conjugated cholic acid and β-muricholic acid as well as hepatic and serum lipids. Additionally, distinct microbiota profiles were found in WD-fed WT mice harboring simple steatosis and CD-fed FXR KO mice, in which the steatosis had a potential to develop into liver cancer. Together, the presented data revealed FXR-dependent concomitant relationships between gut microbiota, BAs, and metabolic diseases in both genders. Gender differences in BAs and microbiota may account for gender dissimilarity in metabolism and metabolic diseases.

## Introduction

Women and men differ substantially regarding the degree of insulin sensitivity, body composition, energy balance, and the incidence of metabolic diseases^1, 2^. The mechanisms accounting for gender difference in metabolism and metabolic disease remain to be established. The liver receives 70% of its blood from the intestine and is constantly exposed to intestinal-derived metabolites. Therefore, gut dysbiosis plays a crucial role in the development of metabolic diseases^3^. Whether gut-derived signaling explains the gender difference in metabolism as well as metabolic diseases remains to be explored. In addition, the pathways linking microbiota and metabolism remain to be fully established. Bile acids (BAs), which are jointly generated by hepatic and bacterial enzymes, play a crucial role in regulating metabolism as well as immune response^4–19^. Thus, microbiota can modulate host metabolism through modification of BAs as well as farnesoid X receptor (FXR) signaling^20–23^. The current study examined the relationship between microbiota and BAs in the development of metabolic syndrome in both genders. We hypothesize that gender differences in metabolic disease are in part due to gender disparity in microbiota and their associated BAs.

About 20–30% of patients who have steatosis develop steatohepatitis, of which only 15–25% progress to fibrosis and cirrhosis^24, 25^. In addition, 80% of obese patients have non-alcoholic fatty liver disease (NAFLD), but up to 16% of lean people also have NAFLD^26^. We question whether all steatosis is identical. In rodents, high-fat diet (HFD) alone induces obesity and steatosis, but not liver cancer. HFD also causes dysregulated BA synthesis. However, FXR knockout (KO) mice, which also have dysregulated BA synthesis, are leaner than wild type (WT) mice, and spontaneously develop steatosis that progresses to steatohepatitis and tumorigenesis when they are about 14 months old^11, 27–30^. In contrast to the whole body FXR KO, liver cancer formation is not noted in organ-specific FXR KO mice^31^. The clinical relevance of FXR KO mice has been revealed based on reduced FXR being found in patients who have liver cirrhosis and cancer^32, 33^. Thus, the current study examined the signaling that may account for progression of metabolic diseases using the whole body FXR KO mouse model.

HFD (36% fat and 14% sugar) is frequently used to study steatosis. Because of the significant role of FXR and BAs in regulating carbohydrate metabolism, it is important to examine the impact of sugar overload in FXR KO mice. In addition, ketogenic HFD actually reduces body weight, which may not be relevant to obesity^34^. Moreover, methionine and choline deficient diet-induced steatosis can lead to liver cancer in rodents, but such diet improves insulin sensitivity^35^. The Western dietary pattern is a meat-sweet diet, which is fat and sugar overloaded. Therefore, for the first time, the design for current study is to allow WT and FXR KO mice of both genders to have a free access to a Western diet (WD, 21% fat and 34% sucrose) that contains more table sugar but less fat compared with HFD to uncover the microbiota and BAs associated with fat as well as sugar overload.

Our results revealed that mice harboring steatosis induced by Western diet intake or FXR deficiency had a distinct gut microbiota profile. WD feeding of FXR KO mice further increased the severity of steatosis, which was worse in male than female mice. Specific BAs and microbiota associated with differential susceptibility to insulin resistance and steatosis were uncovered. In addition, gender differences in insulin sensitivity, WD-induced steatosis, and predicted microbiota functions were FXR-dependent. This study provides novel insights into the prevention and treatment of metabolic diseases by targeting specific microbiota based on gender and disease etiology.

## Results

### FXR deficiency leads to gender difference in WD-induced steatosis

**Figure 1:**
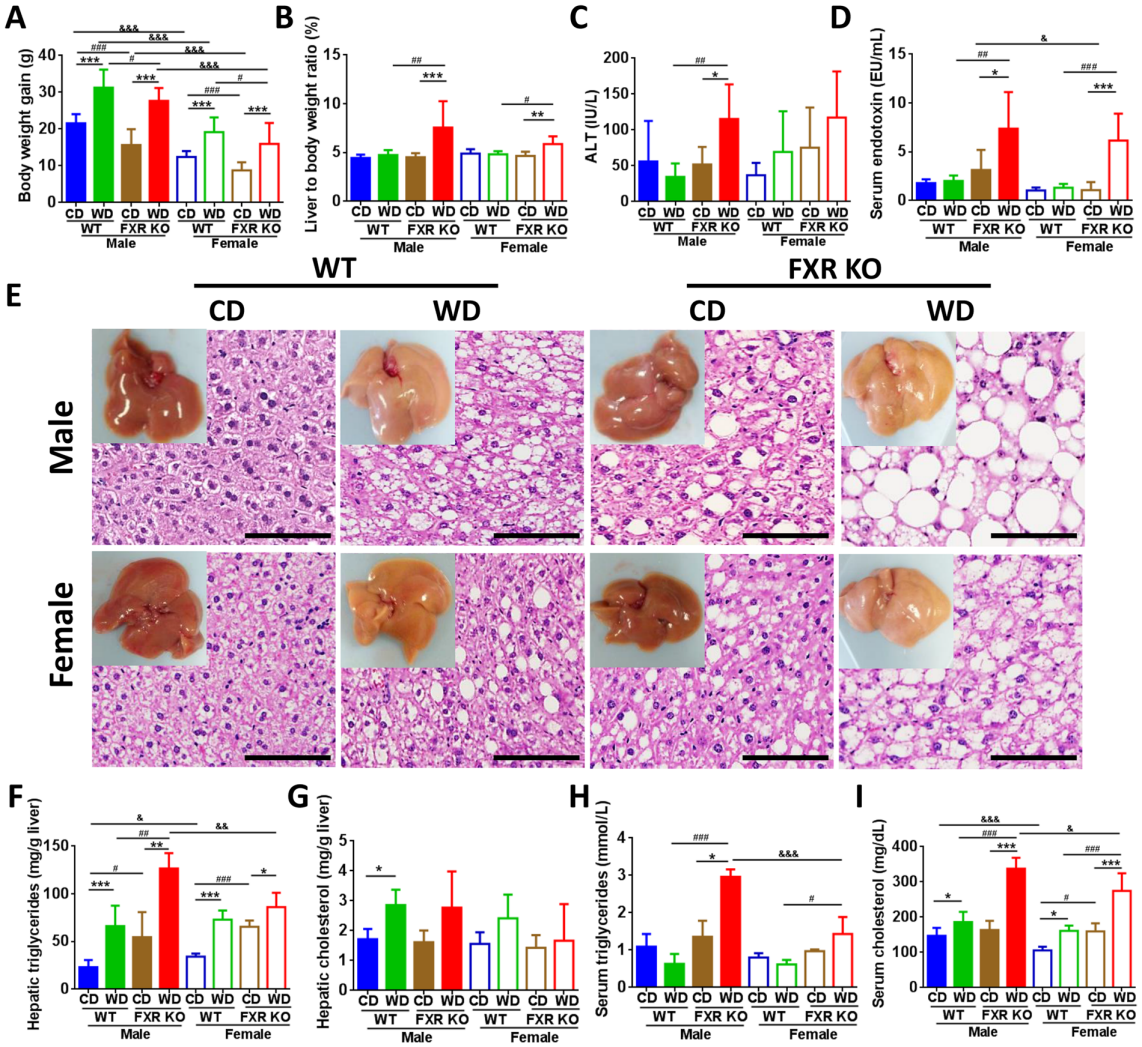
Histological and phenotypic changes in control diet and Western diet-fed wild type and FXR KO mice of both genders. (**A**) Body weight gain. (**B**) Percentage of liver to body weight ratio. (**C**) Serum alanine aminotransferase (ALT). (**D**) Serum endotoxin level. (**E**) Representative liver morphology and H&E-stained liver sections. Scale bars, 100 µm. Hepatic triglycerides (**F**) and cholesterol (**G**) level. Serum triglycerides (**H**) and cholesterol (**I**) level. *n* = 6 per group. Data are expressed as mean ± SD. One-way ANOVA with Tukey’s correction. *p < 0.05, **p < 0.01, ***p < 0.001 for diet comparison; ^#^ *p* < 0.05, ^##^ *p* < 0.01, ^###^ *p* < 0.001 for genotype comparison; ^&^ *p* < 0.05, ^&&^ *p* < 0.01, ^&&&^ *p* < 0.001 for gender comparison.

WD induced body weight gain (Fig. 1A), but FXR KO mice were leaner than their WT counterparts regardless of gender or diet consumed (Fig. 1A). However, FXR deficiency did not affect food intake (Kcal/g body weight/day) (data not shown). Although females were leaner than males, their food intake was about 30% more than the males (data not shown). WD-fed FXR KO mice had elevated liver-to-body weight ratios (hepatomegaly), ALT (alanine aminotransferase), and serum LPS (lipopolysaccharide) (Fig. 1B–D), indicating WD as well as FXR inactivation increased liver injury and inflammatory signaling, which might compromise lipid metabolism.

Both WD and FXR deficiency induced steatosis (Fig. 1E, Supplementary Fig. S1A). Though the degree of steatosis was similar in WD-fed WT mice of both genders, it became more serious in WD-fed FXR KO male mice than the female counterparts indicating gender difference in WD-induced steatosis was FXR-dependent (Fig. 1E, Supplementary Fig. S1A). WD intake and lack of FXR increased hepatic triglyceride levels and male WD-fed FXR KO mice had highest hepatic triglyceride level (Fig. 1F). However, hepatic cholesterol levels were increased by WD intake in WT mice, but not by FXR deficiency (Fig. 1G). Because FXR KO mice had elevated BAs (shown below), it might imply that hepatic cholesterol was quickly converted into BAs. Moreover, WD-fed FXR KO male mice also had the highest serum triglyceride and cholesterol levels (Fig. 1H,I). Ileal histology was not distinctly changed among eight studied groups (Supplementary Fig. S1B). However, the expression levels of ileal genes changed significantly as shown below.

### Gender difference in insulin sensitivity is FXR-dependent

**Figure 2:**
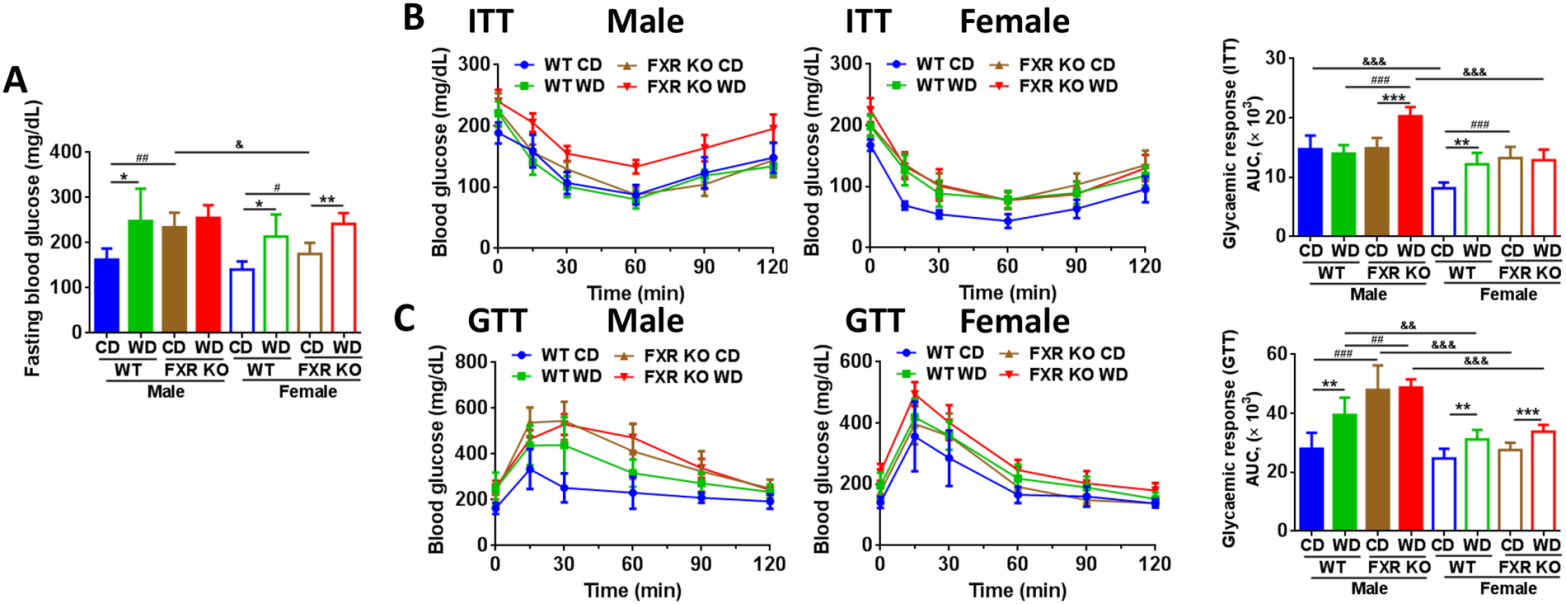
Insulin and glucose tolerance tests in control diet and Western diet -fed wild type and FXR KO mice of both genders. (**A**) Blood glucose level after 6 h fasting. (**B**) Insulin tolerance test (ITT). (**C**) Glucose tolerance test (GTT). The area under curve (AUC) is showed. *n* = 6 per group. Data are expressed as mean ± SD. One-way ANOVA with Tukey’s correction. *p < 0.05, **p < 0.01, ***p < 0.001 for diet comparison; ^#^ *p* < 0.05, ^##^ *p* < 0.01, ^###^ *p* < 0.001 for genotype comparison; ^&^ *p* < 0.05, ^&&^ *p* < 0.01, ^&&&^ *p* < 0.001 for gender comparison.

Although FXR KO mice were much leaner than WT mice, the fasting blood glucose levels were higher in control diet (CD)-FXR KO than WT mice, but they became similar when mice consumed WD (Fig. 2A). Insulin tolerance test (ITT) revealed gender difference in insulin sensitivity was apparent in CD-fed WT mice, but abolished due to FXR deficiency under the same diet (Fig. 2B). However, gender difference in ITT remained in WD-fed FXR KO mice. In addition, WD intake and lack of FXR were equally effective in inducing insulin resistance in females (Fig. 2B). In contrast, among males, increased insulin resistance was only noted when FXR KO mice consumed WD (Fig. 2B). Among eight groups, male WD-fed FXR KO mice were the most insulin resistant. Glucose tolerance test (GTT) results were similar to fasting glucose data (Fig. 2C).

### Differential BA dysregulations induced by diet and FXR deficiency in both genders

**Figure 3:**
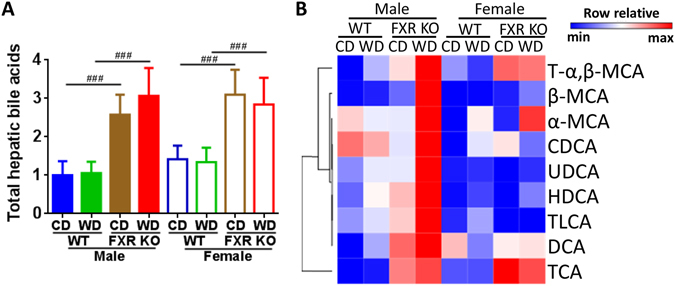
Hepatic bile acid profile in control diet and Western diet-fed wild type and FXR KO mice of both genders. (**A**) Total hepatic bile acids. Data are expressed as mean ± SD. One-way ANOVA with Tukey’s correction, ^###^ *p* < 0.001 for genotype comparison. (**B**) Hepatic bile acid profile. *n* = 16 in male groups, *n* = 6 in female groups.

WD intake shifted hepatic BA profiles, but not total hepatic BA concentration (Fig. 3, Supplementary Table S1). Notably, WD increased the concentration of taurine-conjugated α,β-MCA (T-α,β-MCA) in male mice of both genotypes. The level of α-muricholic acid (α-MCA) and β-MCA was significantly higher in WD than CD-fed FXR KO mice of both genders. In addition, WD-fed FXR KO mice had elevated UDCA (ursodeoxycholic acid), HDCA (hyodeoxycholic acid), and TLCA (taurolithocholic acid) than CD-fed counterparts in male mice.

FXR deficiency significantly increased total hepatic BAs and altered BA profiles (Fig. 3A,B). Moreover, WD-fed FXR KO male mice had the highest concentrations of most BAs, and such changes were accompanied by increased hepatic and serum lipids levels shown in Fig. 1. Hepatic T-α,β-MCA and taurine-conjugated cholic acid (TCA) were consistently elevated due to lack of FXR in both genders (Fig. 3, Supplementary Table S1). Moreover, the concentration of β-MCA and DCA (deoxycholic acid) was increased in male FXR KO in comparison with their WT counterparts. BA profile, but not total hepatic BA, was different between the genders. Hepatic UDCA and HDCA were consistently higher in males than females.

### Gender differences in the expression of lipid and BA homeostasis genes

**Figure 4:**
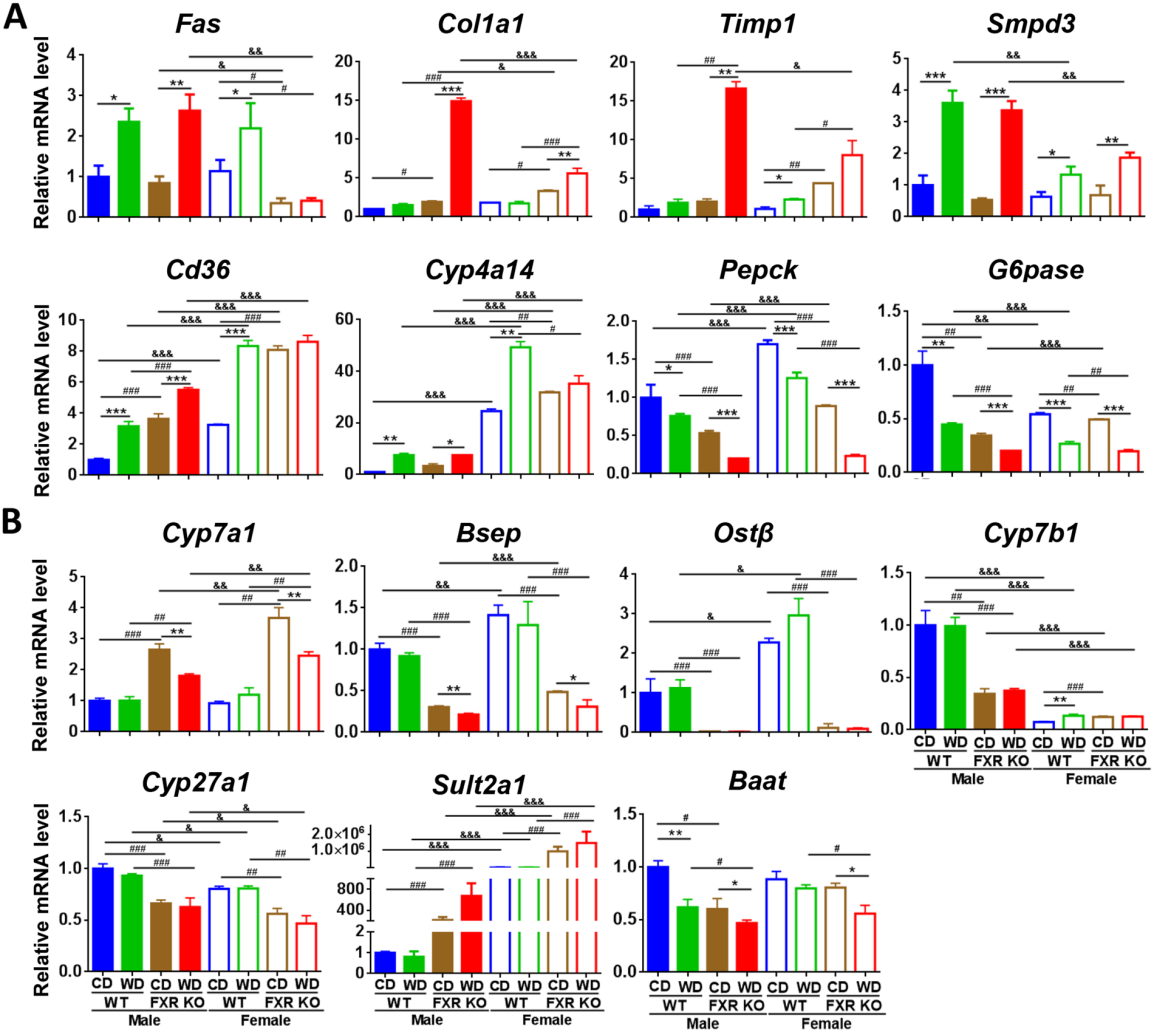
Hepatic gene expression in control diet and Western diet -fed wild type and FXR KO mice of both genders. (**A**) Lipid and glucose related genes. (**B**) Bile acid related genes. *n* = 6 per group. Data are expressed as mean ± SD. One-way ANOVA with Tukey’s correction. *p < 0.05, **p < 0.01, ***p < 0.001 for diet comparison; ^#^ *p* < 0.05, ^##^ *p* < 0.01, ^###^ *p* < 0.001 for genotype comparison; ^&^ *p* < 0.05, ^&&^ *p* < 0.01, ^&&&^ *p* < 0.001 for gender comparison.

Gender difference in expression of lipid related genes was FXR-dependent (Fig. 4A). WD-induced fatty acid synthase (*Fas*) mRNA was not found in female FXR KO mice and gender difference in *Fas* level was apparent in FXR KO mice. Similarly, gender differences in *Col1a1* (collagen type I alpha 1) and *Timp1* (tissue inhibitor of metalloproteinases 1) mRNA levels were apparent in WD-fed FXR KO mice. Ceramides are implicated in the development of steatosis^21^. WD-induced *Smpd3* (sphingomyelin phosphodiesterase 3) expression was also much greater in male than female mice. These findings may explain in part the gender-difference in steatosis found in WD-fed FXR KO mice. In contrast, all 4 groups of female mice had higher fatty acid translocase *Cd36* and fatty acid omega-hydroxylase *Cyp4a14* mRNA levels suggesting rapid lipid uptake as well as oxidation in female mice (Fig. 4A). The expression level of gluconeogenic genes *Pepck* (phosphoenolpyruvate carboxykinase) and *G6Pase* (glucose-6-phosphatase) was also gender different in most cases, but gender disparity was lacking in WD-fed FXR KO mice.

Gender differences in expression of BA homeostasis genes were also noted (Fig. 4B). For example, the level of *Cyp7a1* (cholesterol 7 alpha-hydroxylase) mRNA, which was similar in WT mice of both genders, was increased due to FXR deficiency and was higher in FXR KO females than males. In addition, the expression levels of *Bsep* (bile salt export pump) and *Ostβ* (organic solute transporter β) were higher in female WT mice and reduced in FXR KO mice. The levels of *Cyp7b1* (oxysterol 7α-hydroxylase) and *Cyp27a1* (sterol 27-hydroxylase), which generate CDCA (chenodeoxycholic acid) leading to the production of α- and β-MCA, were higher in males than females. These findings may in part explain elevated concentrations of those free BAs found in males shown in Fig. 3B. In addition, such gender gaps were narrowed due to FXR inactivation. Moreover, *Sult2a1* (bile salt sulfotransferase) level was much higher in females than males suggesting better sulfation-mediated detoxification in females (Fig. 4B). The highly elevated TCA and T-α,β-MCA in FXR KO mice were correlated with increased *Cyp7a1*. In addition, hepatic BA conjugation gene *Baat* (bile acid-CoA:amino acid N-acyltransferase) was reduced by both WD intake and FXR inactivation. Moreover, in the ileum, the expression level of tight junction genes was reduced by WD and FXR deficiency (Supplementary Fig. S2), suggesting the increased intestinal permeability in these mice.

### Divergent gut dysbiosis induced by diet and FXR-deficiency in both genders

**Figure 5:**
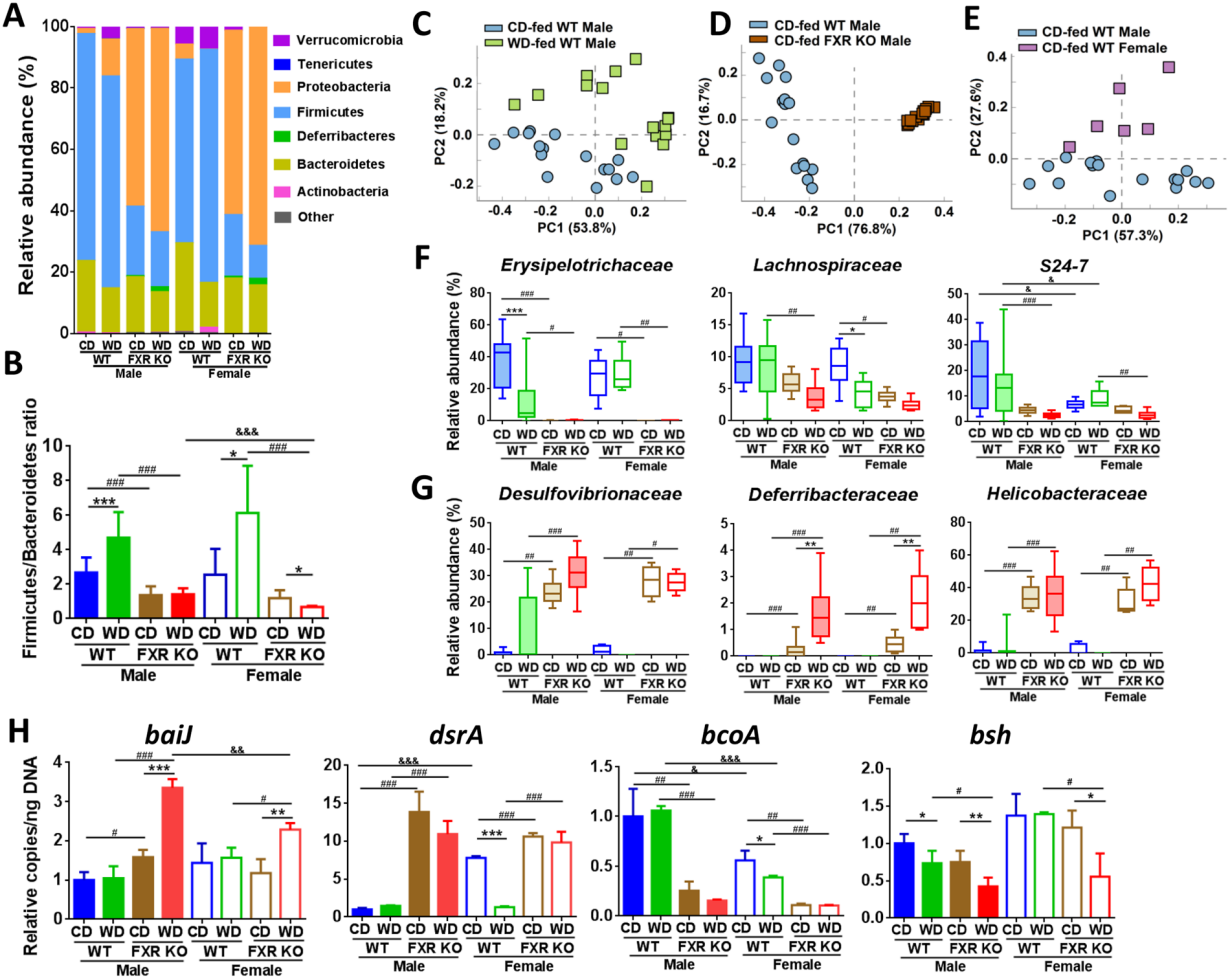
Diet and FXR deficiency changed gut microbiota composition in both genders. (**A**) Cecal microbiota at phylum level. (**B**) Firmicutes to Bacteroidetes ratio. Principal component analysis plots of cecal microbiota at family level based on diet (**C**), phenotype (**D**), and gender difference (**E**). (**F**) and (**G**), relative abundance of cecal microbiota at family level (Kruskal-Wallis test). Box plots display the median, 25th percentile, and 75th percentile; whiskers display minimum and maximum values. (H) Targeted functional quantitative PCR analysis of microbial genes. (**B**,**H**), data are expressed as mean ± SD. One-way ANOVA with Tukey’s correction. *n* = 16 in male groups, *n* = 6 in female groups. *p < 0.05, **p < 0.01, ***p < 0.001 for diet comparison; ^#^ *p* < 0.05, ^##^ *p* < 0.01, ^###^ *p* < 0.001 for genotype comparison; ^&^ *p* < 0.05, ^&&^ *p* < 0.01, ^&&&^ *p* < 0.001 for gender comparison.

WD shifted the gut microbiota in a FXR-dependent manner. The most significant changes caused by FXR deficiency were Firmicutes reduction and Proteobacteria increase (Fig. 5A, Supplementary Table S2). The WD-increased Firmicutes to Bacteroidetes ratio in obese WT mice was not found in leaner FXR KO mice showing FXR dependency (Fig. 5B). Additionally, Firmicutes/Bacteroidetes was reduced due to FXR deficiency. Furthermore, it is apparent that WD-fed WT and CD-fed FXR KO mice acquired distinctive patterns of dysbiosis despite both models generating similar severity of steatosis (Fig. 5A). The shifted microbiota at the family level, which was based on diet, phenotype, and gender, was revealed by principal component analysis (PCA). Examples are shown in Fig. 5C–E and FXR deficiency had the greatest impact. Other comparisons showed similar patterns (data not shown).

Heatmap (Supplementary Fig. S3) and box plots (Fig. 5F,G) revealed that WD altered microbiota at the family level in a gender and FXR-dependent manner. WD-reduced *Erysipelotrichaceae* and *Lachnospiraceae* in WT mice were male and female specific, respectively (Fig. 5F). WD-enriched *Clostridiales_f* and *Clostridiaceae* in WT mice were also male and female specific, respectively (Supplementary Fig. S3B). In addition, such changes were not noted in FXR KO mice. FXR deficiency reduced the abundance of *Erysipelotrichaceae*, *Lachnospiraceae*, *S24*-*7* (Fig. 5F), and increased the abundance of *Desulfovibrionaceae*, *Deferribacteraceae*, and *Helicobacteraceae* (Fig. 5G). The enriched *Deferribacteraceae* in FXR KO mice was further increased by WD intake in both genders. The increase of *Helicobacteraceae* was particularly impressive from 1% in WT mice up to 40% in FXR KO mice (Fig. 5G). Moreover, gender difference was also observed. WT female mice had lower abundance of *S24-7* and higher abundance of *Bacteroidaceae*, *Rikenellaceae*, *Lactobacillaceae*, and *Verrucomicrobiaceae* than male counterparts, but these gender differences were abolished due to FXR deficiency (Supplementary Fig. S3B).

The abundance of cecal bacterial genes was quantified to understand the global microbiota function. FXR inactivation increased the abundance of secondary BA-generating *baiJ* and WD further enhanced it in a male predominant manner (Fig. 5H). Additionally, FXR deficiency also increased the abundance of hydrogen sulfide-producing *dsrA* while substantially reducing butyrate-producing *bcoA*. The abundance of BA de-conjugation gene *bsh* was reduced by both WD and FXR deficiency, which was consistent with increased conjugated BAs *in vivo* (Fig. 5H).

To study the potential function of gut microbiota, PICRUSt (Phylogenetic Investigation of Communities by Reconstruction of Unobserved States) and LEfSe analysis (Linear discriminant analysis Effect Size) were performed to predicate and identify differentially enriched pathways. The predicted microbiota functions shifted based on 3 variables as summarized in Table 1. According to the number of pathways differentially enriched between groups (LDA score >2, *p* < 0.05), FXR KO had the greatest impact, consistent with the PCA finding shown in Fig. 5D. There were 93–161 pathways differentially enriched between WT and FXR KO groups. Regarding diet, there were 19 pathways differentially enriched between CD and WD-fed male WT mice, but only 4 in FXR KO mice. In addition, diet had zero impact on changing the function of microbiota in female WT, but the effect was substantially increased in female KO mice (0 *vs*. 45 pathways) indicating FXR-dependency in regulating gut microbiota function in response to dietary challenge. Furthermore, WD enriched neurodegenerative disease pathways in female FXR KO mice indicating gender difference in the gut-brain axis affected by dysregulated BAs and dysbiosis. It is important to note that gender difference in microbiota function was abolished by FXR deficiency, and there was no differentially enriched pathway between male and female FXR KO mice (Table 1). Gender differences became very apparent when both genotypes of mice consumed WD consistent with the gender difference in steatosis.

**Table 1:** Differentially enriched microbial pathways in each group.

Group	Number of pathway, examples of differentially enriched pathways in each group				Total number
**Diet**		**Control diet**		**Western diet**	
Male WT	15	• Amino sugar and nucleotide sugar metabolism	4	• C5-Branched dibasic acid metabolism	19
		• Photosynthesis		• Naphthalene and ethylbenzene degradation	
		• Alanine-Aspartate and glutamate metabolism		• Cyanoamino acid metabolism	
Male FXR KO	2	• Methane metabolism	2	• Photosynthesis	4
		• Lipoic acid metabolism		• Vibriocholerae pathogenic cycle	
Female WT	0		0		0
Female FXR KO	20	• Starch and sucrose metabolism	25	• Bacterial motility and flagellar assembly	45
		• Galactose metabolism		• Fatty acid biosynthesis	
		• Glycolysis and gluconeogenesis		• Neurodegenerative Diseases (Parkinson’s, Huntington’s, Alzheimer’s disease)	
**Genotype**		**Wild type**		**FXR KO**	
CD-fed Male	29	• Transporter	64	• Lipopolysaccharide biosynthesis	93
		• Fructose and mannose metabolism		• Fatty acid biosynthesis	
		• Fatty acid metabolism		• Neurodegenerative Diseases	
WD-fed Male	37	• Transporter	74	• Lipopolysaccharide biosynthesis	111
		• Transcription factors		• Carbon fixation pathway in Prokaryotes	
		• Fructose and mannose metabolism		• Bacterial secretion system	
CD-fed Female	53	• Phosphotransferase system	48	• Bacterial motility proteins	101
		• Amino sugar and nucleotide sugar metabolism		• Flagellar assembly	
		• Starch and sucrose metabolism		• Secretion system	
WD-fed Female	85	• Transporter	76	• Lipopolysaccharide biosynthesis	161
		• Transcription factors		• Bacterial motility proteins	
		• Fructose and mannose metabolism		• Carbon fixation pathway in Prokaryotes	
**Gender**		**Male**		**Female**	
CD-fed WT	4	• Bacterial motility and flagellar assembly	7	• G-protein coupled receptors	11
		• Bacterial chemotaxis		• Glycerophospholipid metabolism	
		• Plant pathogen interaction		• Nucleotide excision repair	
WD-fed WT	8	• Methane metabolism	33	• Replication recombination and repair	41
		• Starch and sucrose metabolism		• DNA repair and recombination	
		• Cyanoamino acid metabolism		• Fatty acid metabolism	
CD-fed FXR KO	0		0		0
WD-fed FXR KO	23	• Starch and sucrose metabolism	34	• Retinoic acid-inducible gene like receptor signaling pathway	57
		• Methane metabolism		• Bacterial secretion system	
		• Glycolysis and gluconeogenesis		• Metabolism of cofactors and vitamins	

### Integrated relationships

**Figure 6:**
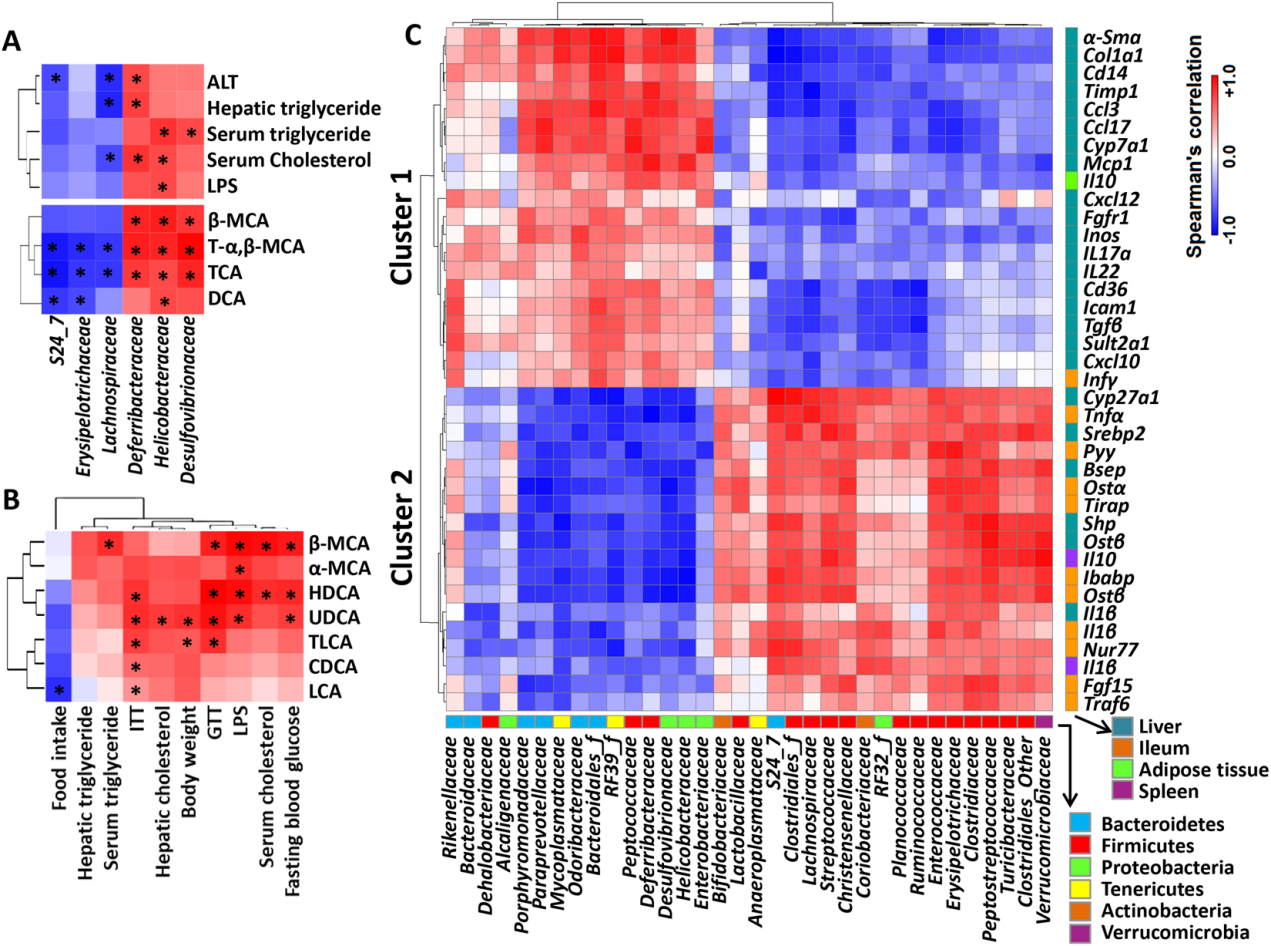
Spearman’s correlation analysis. Heatmaps of Spearman’s correlation analysis between abundance of bacterial families and phenotypes, bacterial families and hepatic bile acids (**A**), between mouse phenotypes and hepatic bile acids (**B**), and between abundance of bacterial families and genes (**C**) *p < 0.05.

The relationships between gut microbiota, hepatic BAs, mouse phenotypes, and gene expression were established. Spearman’s correlations revealed that 20 out of 34 identified bacterial families had strong correlations with certain mouse phenotypes (Supplementary Fig. S4). ALT, hepatic triglyceride and serum cholesterol were negatively correlated with *Lachnospiraceae* and positively correlated with *Deferribacteraceae* (Fig. 6A). Moreover, *Helicobacteraceae* were positively correlated with serum triglyceride, cholesterol, and LPS (Fig. 6A).

In addition, 25 out of 34 identified bacterial families had strong correlations with certain BAs (Supplementary Fig. S5). The concentrations of T-α,β-MCA and TCA were negatively correlated with the abundance of *S24-7*, *Erysipelotrichaceae*, and *Lachnospiraceae*. T-α,β-MCA, TCA, β-MCA, and DCA were positively associated with the abundance of *Deferribacteraceae*, *Helicobacteraceae*, and *Desulfovibrionaceae* in most cases (Fig. 6A).

Between BAs and phenotypes (Supplementary Fig. S6), the male predominant UDCA and HDCA were positively associated with blood glucose levels based on GTT and ITT (Fig. 6B). In addition, UDCA, β-MCA, and HDCA, of which were elevated in males and/or FXR KO, were positively correlated with LPS. Most of those BAs showed positive correlation with ITT (Fig. 6B).

Spearman’s correlations also revealed relationships between microbiota abundance and 163 studied host genes (Supplementary Fig. S7). Those genes have known roles in regulating BA homeostasis, metabolism, inflammatory process, cell cycle progression, etc. in the liver, ileum, spleen, and adipose tissue. There were two distinct clusters of clearly associated specific bacterial families (Fig. 6C, Supplementary Fig. S7). In cluster 1, majority were hepatic genes implicated in inflammation or fibrosis (*α-Sma*, *Col1a1*, *Cd14*, *Timp1*, *Ccl3*, *Ccl17*, *Mcp1*, *Cxcl12*, *Inos*, *Il17a*, *Il22*, *Icam1*, *Tgfβ*, and *Cxcl10*), BA homeostasis (*Cyp7a1* and *Sult2a1*), and energy and fat homeostasis (*Fgfr1* and *Cd36*). Cluster 2 are genes in regulating BA homeostasis (hepatic *Cyp27a1*, *Bsep*, *Shp*, *Ostβ*, and ileal *Ostα*, *Ostβ*, *Ibabp*, *Fgf15*), energy homeostasis (hepatic *Srebp2*, ileal *Pyy* and *Nur77*), and inflammation (ileal *Tnfα*, *Tirap*, *Traf6*, *Il1β*, spleen *Il10*, *Il1β*, and hepatic *Il1β*) (Fig. 6C). Three main bacterial phyla, Bacteroidetes, Firmicutes, and Proteobacteria, were clustered separately. Six families under Bacteroidetes and four families under Proteobacteria including *Desulfovibrionaceae* and *Helicobacteraceae* showed a positive and negative relationship with Cluster 1 and 2, respectively. By contrast, many families within Firmicutes, including FXR KO-reduced *Lachnospiraceae* and *Erysipelotrichaceae*, were positively correlated with the expression levels of Cluster 2 genes, but negatively associated with Cluster 1 genes.

### Divergent BA profile and gut dysbiosis in Western diet and FXR deficiency-induced steatosis

**Figure 7:**
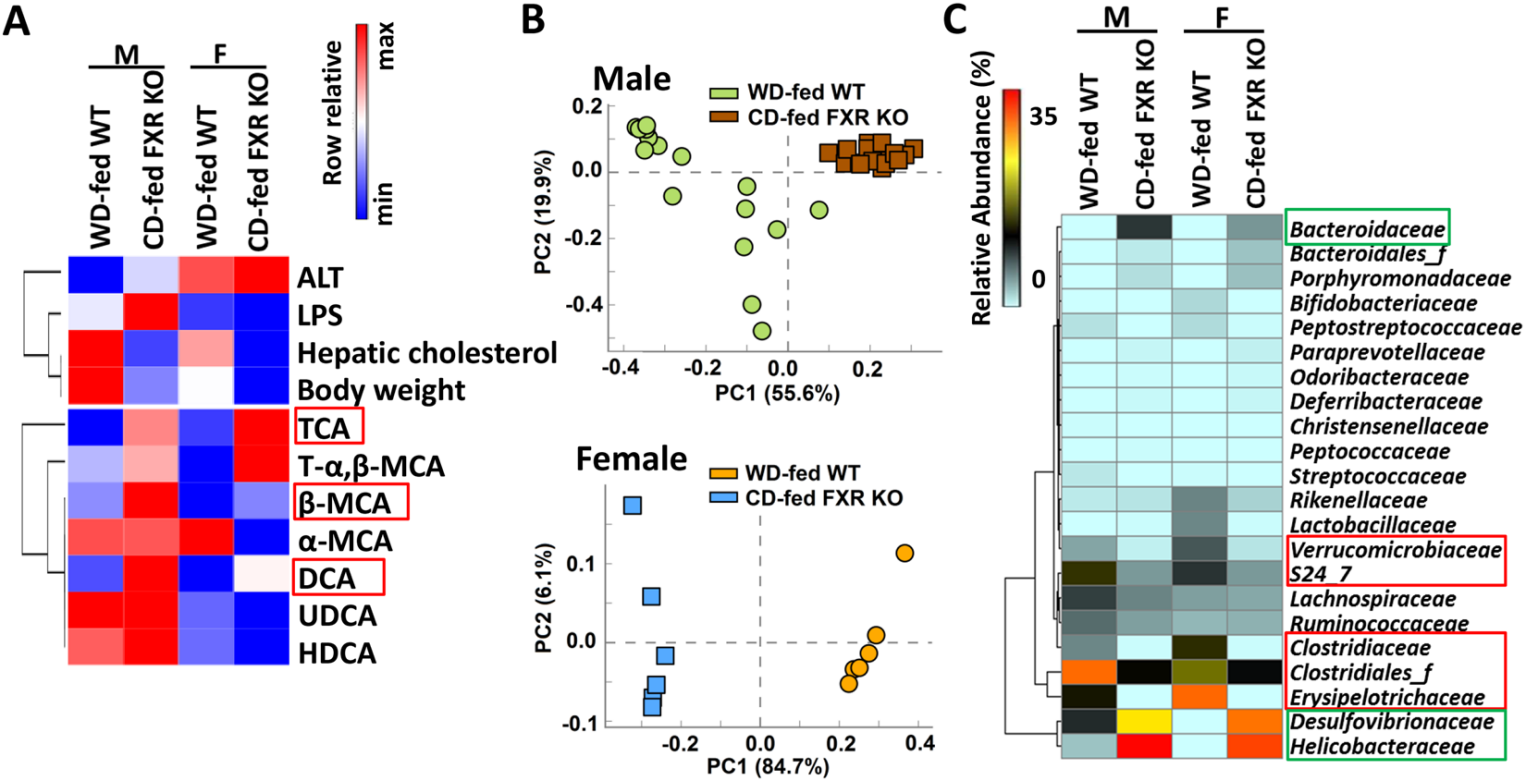
The differences between Western diet-fed wild type mice and control diet-fed FXR KO mice of both genders. (**A**) Heatmaps of bile acid profile and mouse phenotypes. Bile acids and phenotypes were displayed with fold change (WD-fed WT *vs*. CD-fed FXR KO) ≥1.5 or ≤0.67 in at least one gender. (**B**) Principal component analysis plots of cecal microbiota at family level. (**C**) Bacterial families are shown (mean relative abundance >0.2%) with significant difference between WD-fed WT and CD-fed FXR KO mice in at least one gender.

Histologically, the steatosis induced by WD and FXR deficiency was similar (Fig. 1E, Supplementary Fig. S1). However, aged FXR KO mice spontaneously develop liver tumors^11, 27–30^. To define the difference, comparisons were done between WD-fed WT and CD-fed FXR KO mice of both genders. Despite having higher hepatic cholesterol as well as more body weight gain, WD-fed WT mice had lower hepatic TCA, DCA, and β-MCA, in both genders showing inferior effects of lacking FXR (Fig. 7A, Supplementary Table S3). Gut microbiota was also strikingly different between the two models. The PCA plots showed distinct clustering of WD-fed WT and CD-fed FXR KO at the bacterial family level in both genders (Fig. 7B). The abundances of *Verrucomicrobiaceae*, *S24-7*, *Clostridiaceae*, *Clostridiales_f*, and *Erysipelotrichaceae* were much higher in WD-fed WT mice, while *Bacteroidaceae*, *Desulfovibrionaceae*, and *Helicobacteraceae* were markedly higher in CD-fed FXR KO mice of both genders (Fig. 7C).

## Discussion

Gender disparity in metabolism and metabolic disease has been long thought to be regulated intrinsically by the host. Our study revealed BAs and FXR as well as associated gut microbiota are implicated in the gender difference in metabolic diseases. By comparing WD and FXR KO-induced steatosis, our data revealed specific microbiota families and BAs were differentially responsible for obesity versus metabolic dysregulation-associated steatosis that has the potential for tumorigenesis. The data presented suggest the use of probiotics or other means in the prevention and treatment of metabolic disease should be tailored based on gender and other phenotypes such as microbiota and BA profiles.

The detrimental effects of BAs over a lifetime of exposure vary depending on their solubility, conjugation status, and bioactivity to their receptors. Without self-regulatory mechanisms controlled by FXR, exposure to high concentration of BAs may cause cholestasis, inflammation, and tumorigenesis^6–8^. Among the identified BAs, hepatic free β-MCA concentration was extremely low in CD-fed WT mice, but showed the most substantial fold increase as a result of WD intake, FXR deficiency, or being male (up to 15 folds). The extent of induction was positively associated with detrimental outcomes including LPS, glucose intolerance as well as serum lipids. WD-fed FXR KO male mice, which exhibited the worst pathological phenotype, had the highest hepatic β-MCA level. It is important to note that β-MCA is also detectable in humans^36^. Hepatic TCA concentration was increased due to FXR deficiency, but not WD intake. This finding was consistent with the finding that TCA concentration is reduced by FXR agonist (obeticholic acid) treatment^4^. In addition, TCA is elevated in nonalcoholic steatohepatitis (NASH) patients^37, 38^. Due to the affinity of TCA to FXR and TGR5, it is possible that the elevated TCA is trying to activate these receptors in order to normalize metabolism indicating a compensatory effect. Moreover, TCA treatment inhibited intestinal adenoma formation in mice^39^ and increased the circulating GLP-1, PYY, and insulin, thereby decreasing glucose levels in obese type 2 diabetic patients^40^. Consistently, our data showed that the elevated hepatic TCA was not significantly associated with negative outcomes including increased ALT, serum and hepatic triglycerides, LPS, as well as glucose level suggesting there was no negative impact for TCA induction in FXR KO mice (Supplementary Fig. S6). Male-predominant UDCA and HDCA were positively correlated with serum LPS, glucose intolerance, and insulin resistance. Elevated serum UDCA was also found in patients with alcoholic liver and biliary tract disease^41^. It would be important to further elucidate the specific effects of those BAs at relevant concentrations found *in vivo*. Taken together, FXR KO mice had obvious phenotypes on both genders and WD intake enhanced the gender differences.

Both WD used in the current study and HFD reduced the abundance of *Bacteroidaceae* and *Erysipelotrichaceae* ^42, 43^. However, WD increased *Clostridiales_f*, while HFD reduced it in males^42^. In addition, HFD, but not WD, enriched the abundance of *Ruminococcaceae* and *Rikenellaceae* in males^42^. The WD we used had more carbohydrates but less fat compared with HFD. The impact of carbohydrates in metabolic disease process remains to be delineated.

Gut microbiota is correlated with the development of obesity. However, conflicting findings persists regarding the relationship of obesity and the relative proportions of Firmicutes and Bacteroidetes in mice and humans studies^44–47^. Our data showed WD-fed obese WT mice had increased Firmicutes to Bacteroidetes ratio than CD-fed lean mice in both genders. In addition, obese WT mice had higher Firmicutes/Bacteroidetes than the leaner FXR KO mice. However, WD induced Firmicutes/Bacteroidetes was FXR dependent. Moreover, although male mice gained more body weight than females, the correlated ratio change was not noted. Thus, the Firmicutes/Bacteroidetes ratio can be affected by metabolic condition and gender.

FXR has an essential role in regulating gut microbiota profile. Based on methods used, differences of microbiota composition in WT and FXR KO mice were reported between aerobic *vs*. anaerobic bacteria or based on selected phyla and families^20, 23, 48, 49^. Moreover, FXR deficiency abolished probiotic-mediated alternation of BA metabolism indicating the close relationship between FXR signaling pathway and gut microbiota^48^. The current study might be one of the first to compare the dietary effect in both genders based on FXR status. Our data showed that the abundances of Proteobacteria (from 2% to 58%) and Firmicutes (from 74% to 23%) had the largest changes due to FXR deficiency. Moreover, Proteobacteria was further increased by WD intake in FXR KO mice. Progressive increases in the abundance of Proteobacteria were also found in children who are healthy, obese, or have NASH^50^. The reduced abundance of *Erysipelotrichaceae* (from 37% to 0.1%) and *Lachnospiraceae* (from 10% to 6%) families can account for the substantial reduction of Firmicutes (from 74% to 23%) in FXR KO mice. Higher abundance of *Erysipelotrichaceae* was found in morbidly obese individuals^44^. Many *Lachnospiraceae* species generate butyrate, which regulates metabolism, immune response, and colonocyte growth^51^. Consistently, our data showed reduced abundance of butyrate-producing gene (*bcoA*) in the cecal content of FXR KO mice. *Lachnospiraceae*, which was reduced in colorectal cancer patients^52^, was negatively correlated with ALT, hepatic triglyceride, and serum cholesterol in current study.

*Desulfovibrionaceae* (from 1% to 24%), *Helicobacteraceae* (from 1% to 33%) as well as *Deferribacteraceae* had the largest increases due to FXR deficiency. *Bilophila*, the major genus in the *Desulfovibrionaceae* family, generates hydrogen sulfide, a genotoxin and mucosal barrier-breaker. Consistently, the abundance of cecal hydrogen sulfide-producing gene *dsrA* was elevated in male FXR KO mice. *Helicobacteraceae* generates LPS, and its abundance is correlated with hepatic DCA that has DNA damaging, pro-inflammatory, and tumor-promoting effects^53^. *Helicobacteraceae* family is implicated in various health issues and was positively correlated with serum triglyceride and cholesterol in our study. Moreover, the expansion of *Deferribacteraceae* may lead to mucin deregulation, inflammation, and tumorigenesis in FXR KO. The correlation of ALT, hepatic triglyceride, and serum cholesterol with *Deferribacteraceae* was diametrically opposed to *Lachnospiraceae*, suggesting the reverse effect of these two families. Furthermore, the clustered *Desulfovibrionaceae*, *Deferribacteraceae*, and *Helicobacteraceae* were positively correlated with β-MCA, T-α,β-MCA, and TCA, which together constituted 95% of the total hepatic BA pool. In addition, the abundances of those three families were positively correlated with hepatic *Cyp7a1* expression and negatively correlated with *Shp*, *Bsep*, and *Ostβ* expression, which might account for the accumulation of hepatic BAs in FXR KO mice.

Together, gender differences in metabolism are FXR-dependent. For examples gender difference in WD-induced steatosis became apparent when FXR was inactivated. However, gender differences in insulin sensitivity and predicted gut microbial function were not found in FXR KO mice. Moreover, many genes regulating BA homeostasis or metabolism displayed a gender-dependent expression patterns, and such differences became either more apparent or abolished due to FXR deficiency. Furthermore, WD-induced dysbiosis as well as the predicted microbial functions were also gender and FXR-dependent. It is important to emphasize that FXR inactivation, dysregulated BA synthesis, and dysbiosis are tightly linked. It is not possible to determine which has a more significant role in steatosis development. It has been shown that cholestyramine treatment reduces the malignant hepatic lesions in FXR KO mice indicating the significant role of BAs in liver pathology^27^. Additionally, antibiotic modification of gut microbiota prevents HFD-associated NAFLD and alters BA profiles^21^. Therefore, the presented data revealed the concomitant relationships between gut microbiota, BAs, and metabolic diseases in both genders in a FXR-dependent manner.

In conclusion, it is likely that gender difference in FXR signaling accounts for gender disparity in metabolism as well as metabolic disease process. Emerging evidence reveals the significant role of gut microbiota in contributing to gender difference in immunity^54^. The interactions between FXR and sex hormones warrant further investigation. It would be critically important to study gender-specific effects controlled by gut microbiota in order to develop tailored prevention and treatment strategy using probiotics for metabolic diseases.

## Methods

### Mice

C57BL/6 WT (Jackson Laboratory) and FXR KO^55^ mice of both genders were given a WD (21% fat, 34% sucrose, and 0.2% cholesterol, w/w) or CD (5% fat, 12% sucrose, and 0.01% cholesterol, w/w) from Harlan Teklad after weaning (3 weeks) and were euthanized at the age of 5 months. Experiments were conducted in accordance with the National Institutes of Health Guide for the Care and Use of Laboratory Animals under protocols approved by the Institutional Animal Care and Use Committee of the University of California, Davis.

### Glucose and insulin tolerance test

After fasting 6 h, tail vein blood was used to establish fasting blood glucose levels. Glucose (2 g/kg body weight) and insulin (0.75 U/kg body weight, Sigma-Aldrich) were given for conducting glucose (GTT) and insulin (ITT) tolerance tests, respectively. Glucose levels were measured 15, 30, 60, 90, and 120 min post treatment by the OneTouch Ultra 2 (Johnson). The area under the curve (AUC) of the blood glucose levels over time was calculated.

### Biochemical analysis

Serum alanine aminotransferase (ALT, Pointe Scientific), lipopolysaccharide (LPS, Thermo Scientific), serum and hepatic triglyceride and cholesterol (BioAssay Systems) levels were quantified according to the manufacturer’s instructions.

### Quantification of bile acids

Liver tissue (50–100 mg) was collected from fed-state mice with gallbladder removed following by BA extraction^56^. BAs were analyzed using a Prominence^TM^ UFLC system (Shimadzu) coupled to an API 4000 QTRAP^TM^ mass spectrometer (AB Sciex) operated in the negative ionization mode. Chromatography was performed on a Kinetex C_18_ column (50 mm × 2.1 mm, 2.6 μm) maintained at 40 °C preceded by a high pressure column prefilter. The mobile phase consisted of methanol gradient delivered at a flow rate of 0.4 ml/min^5^.

### Gene expression profiling

Tissue RNA was extracted using TRIzol Reagent (Invitrogen). cDNA was synthesized using High Capacity RNA-to-cDNA Kit (Applied Biosystems). qRT-PCR was performed on an ABI 7900HT Fast real-time PCR system using Power SYBR® Green PCR Master Mix (Applied Biosystems). Primers were designed using Primer3 Input Software (v0.4.0) and the sequences are available upon request. mRNA levels were normalized to the level of *Gapdh* mRNA.

### Quantification of bacterial DNA and 16S rRNA gene sequencing

Cecal (0.1 gram) DNA was extracted using ZR Fecal DNA MiniPrep Kit (Zymo Research), quantified by NanoDrop (NanoDrop Technologies), and amplified using primers based on published sequences (Supplementary Table S4). A dissociation step was included to analyze the melting profile of amplified products. In parallel, qPCR was done using ten-fold serial diluted synthetic DNA fragments of bacterial gene with known concentrations. Bacterial DNA concentration was calculated using standard curves of diluted synthetic DNA fragment.

Pyrosequencing of Tagged 16S rRNA Gene Amplicons of cecal DNA was done based on published methods^57^. The V4 region of 16S rRNA gene was amplified and sequenced using Illumina MiSeq.

### Bioinformatics and statistical analysis

Sequence reads were analyzed using QIIME software. Family-level taxa abundances were used in Statistical Analysis of Metagenomic Profiles (STAMP v.2.0) software to generate principal component analysis (PCA) plots. Spearman’s correlations were performed with R Statistical Software. Functional profiles of microbial communities were predicted using PICRUSt (Phylogenetic Investigation of Communities by Reconstruction of Unobserved States). LEfSe analysis (Linear discriminant analysis Effect Size) was performed to identify differentially enriched pathways^58^. Data are expressed as mean ± SD. Differences between groups in microbiota family level were calculated by Kruskal-Wallis test. All other comparisons were calculated by two-tailed Student’s *t* test or one-way ANOVA followed by Tukey’s test using GraphPad Prism 6 software. *p*-values are adjusted for multiple comparison using false discovery rate. *p* < 0.05 was considered statistically significant.

## Electronic supplementary material

Supplementary information
