# Supplementary material for: Gender Differences in Bile Acids and Microbiota in Relationship with Gender Dissimilarity in Steatosis Induced by Diet and FXR Inactivation
Source: Sci Rep. 2017 May 11;7:1748. doi: 10.1038/s41598-017-01576-9 (PMC5431816; doi:10.1038/s41598-017-01576-9)
Supplement: Supplementary file 1 — Supplementary information [file 41598_2017_1576_MOESM1_ESM.pdf]

Gender Differences in Bile Acids and Microbiota in Relationship with Gender Dissimilarity in Steatosis Induced by Diet and FXR Inactivation

Lili Sheng, Prasant Kumar Jena, Hui-Xin Liu, Karen M. Kalanetra, Frank J. Gonzalez, Samuel W. French, Viswanathan V. Krishnan, David A. Mills, Yu-Jui Yvonne Wan

Table of contents

Supplementary Tables……………………………………………………….…………………......2

Supplementary Figures……………………………………………………………………...……7

Supplementary References……………………………………………….………..……….14

Supplementary Tables

Supplementary Table S1. The concentration (μM) or ratio of hepatic bile acid in CD- and WD-fed WT and FXR KO mice of both genders.

Bile acid	Male								Female							
	WT				FXR KO				WT				FXR KO			
	CD		WD		CD		WD		CD		WD		CD		WD	
	Ave	SD	Ave	SD	Ave	SD	Ave	SD	Ave	SD	Ave	SD	Ave	SD	Ave	SD
TCA	26.221	6.938	29.944	8.420	89.964	16.165	99.931	5.955	40.000	11.883	39.383	7.245	111.400	17.011	98.900	17.458
α-MCA	0.327	0.087	0.288	0.072	0.286	0.071	0.446	0.074	0.152	0.050	0.309	0.090	0.172	0.049	0.416	0.152
CDCA	0.009	0.002	0.008	0.002	0.006	0.002	0.011	0.002	0.002	0.001	0.006	0.002	0.007	0.001	0.004	0.004
UDCA	0.015	0.004	0.023	0.003	0.023	0.003	0.047	0.004	0.005	0.002	0.007	0.003	0.003	0.002	0.007	0.003
HDCA	0.018	0.005	0.037	0.009	0.044	0.010	0.066	0.030	0.007	0.007	0.014	0.007	0.006	0.009	0.021	0.005
TLCA[a]	0.032	0.010	0.047	0.016	0.065	0.027	0.107	0.039	0.009	0.005	0.035	0.013	NA	NA	NA	NA
DCA	0.037	0.010	0.065	0.012	0.104	0.023	0.121	0.008	0.090	0.017	0.058	0.010	0.082	0.032	0.084	0.025
β-MCA	0.366	0.097	0.617	0.117	1.352	0.262	5.360	1.180	0.308	0.129	0.335	0.101	0.598	0.164	1.540	0.536
LCA	0.004	0.001	0.003	0.001	0.003	0.001	0.003	0.001	0.002	0.001	0.003	0.001	0.003	0.001	0.003	0.001
T-α,β-MCA	19.293	5.105	27.369	4.266	32.093	4.177	41.813	4.381	25.932	2.955	21.767	5.331	37.417	2.870	36.550	8.987
TCDCA	2.776	1.651	2.746	0.722	3.074	1.361	2.984	1.634	2.927	1.026	3.920	1.142	2.683	0.761	2.365	0.389
CA	0.190	0.050	0.161	0.042	0.159	0.041	0.262	0.043	0.228	0.029	0.269	0.053	0.254	0.028	0.172	0.089

[a]: TLCA was under the limit of detection in CD- and WD-fed FXR KO female mice.

Supplementary Table S2. Relative abundance (%) of cecal microbiota in CD- and WD-fed WT and FXR KO mice of both genders at phylum level.

Phylum	Male								Female							
	WT				FXR KO				WT				FXR KO			
	CD		WD		CD		WD		CD		WD		CD		WD	
	Ave	SD	Ave	SD	Ave	SD	Ave	SD	Ave	SD	Ave	SD	Ave	SD	Ave	SD
Actinobacteria	0.36	0.46	0.09	0.11	0.03	0.10	0.13	0.47	0.06	0.10	1.76	1.40	0.00	0.00	0.00	0.00
Bacteroidetes	23.33	10.59	14.65	10.65	18.16	5.11	13.20	4.14	28.95	11.69	14.60	6.28	17.98	4.20	15.67	4.06
Deferribacteres	0.00	0.00	0.00	0.00	0.35	0.43	1.58	0.80	0.00	0.00	0.00	0.00	0.53	0.45	2.13	1.11
Firmicutes	73.88	9.15	69.05	14.64	22.62	5.27	17.95	5.08	59.84	10.86	75.95	5.96	20.08	3.43	10.74	3.43
Proteobacteria	1.70	2.22	12.13	16.72	57.88	6.50	66.25	9.07	4.91	2.69	0.05	0.04	60.09	4.52	71.13	8.41
Tenericutes	0.00	0.00	0.08	0.08	0.05	0.09	0.01	0.01	0.00	0.00	0.00	0.00	0.01	0.01	0.00	0.01
Verrucomicrobia	0.73	0.77	4.00	5.91	0.71	0.62	0.68	0.43	5.81	3.85	7.47	1.69	1.31	0.88	0.28	0.27
Other	0.00	0.00	0.00	0.00	0.20	0.18	0.20	0.12	0.43	0.69	0.16	0.11	0.01	0.01	0.06	0.09

Supplementary Table S3. Fold change of phenotypes and hepatic bile acids in Western diet-fed wild type mice and control diet-fed FXR KO mice of both genders.

Phenotype	WD-fed WT *vs.* CD-fed FXR KO	
	**Male**	**Female**
ALT	0.67*	0.92
Hepatic cholesterol	1.77*	1.69*
LPS	0.65*	1.21
Body weight	2.00*	2.18*
**Bile acid**		
TCA	0.33*	0.35*
T-α,β-MCA	0.85	0.58*
α-MCA	1.01	1.80*
UDCA	1.04	2.79*
HDCA	0.85	2.12*
DCA	0.63*	0.70
β-MCA	0.46*	0.56*
LCA	1.07	0.96
TCDCA	0.89	1.46
CA	1.01	1.06
CDCA	1.41	0.97

TLCA[a]	0.72	NA

*: Fold change ≥1.5 or ≤0.67, and *p*<0.05.

[a]: TLCA was under the limit of detection in CD-fed FXR KO female mice.

Supplementary Table S4. List of primers used in this study to determine functional microbial genes.

**Target genes and primers**	**Sequence (5' – 3')**	**Annealing temperature**	**Reference**
Functional gene for secondary bile acids			
baiJF	TCAGGACGTGGAGGCGATCCA	60ºC	[1]
baiJR	TACRTGATACTGGTAGCTCCA		
Functional gene for butyrate producer			
bcoAF	GCIGAICATTTCACITGGAAYWSITGGCAYATG	60ºC	[2, 3]
bcoAR	CCTGCCTTTGCAATRTCIACRAANGC		
Functional gene for hydrogen sulfide			
dsrAF	GCCGTTACTGTGACCAGCC	60ºC	[4]
dsrAR	GGTGGAGCCGTGCATGTT		
Functional gene for BA de-conjugation			
bshF	ATGGGCGGACTAGGATTACC	54ºC	[5,6]
bshR	TGCCACTCTCTGTCTGCATC		

Supplementary Figures

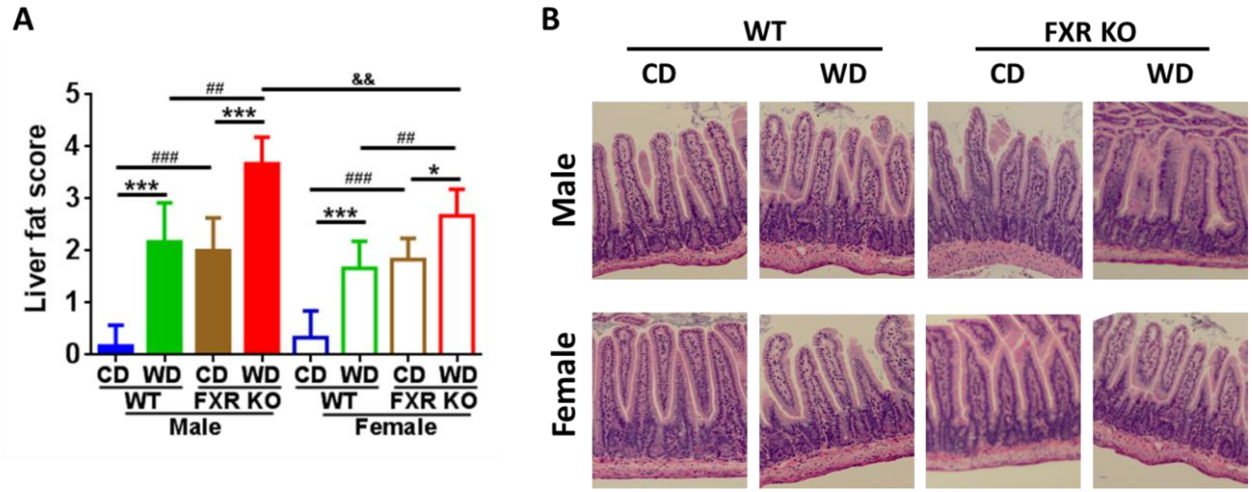

Fig. S1.Liver fat score (A) and representative H&E-stained ileum sections (B). $n = 6$ per group. Data are expressed as mean ± SD. One-way ANOVA with Tukey's correction. * $p<0.05$, ** $p<0.01$, *** $p<0.001$ for diet comparison; $^{\#}p<0.05$, $^{\#\#}p<0.01$, $^{\#\#\#}p<0.001$ for genotype comparison; $^{\&}p<0.05$, $^{\&\&}p<0.01$, $^{\&\&\&}p<0.001$ for gender comparison. Magnification, ×40.

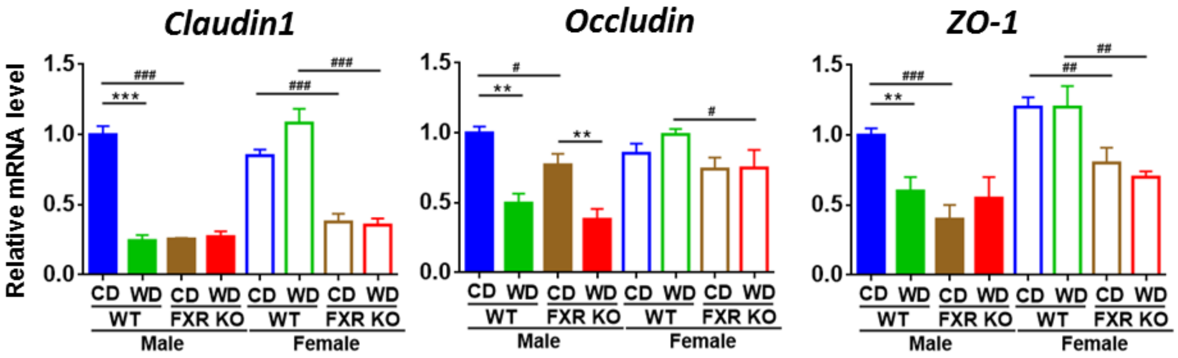

Fig. S2. Tight junction gene expression in the ileum. * p<0.05, ** p<0.01, *** p<0.001 for diet comparison; $^{\#}$ p<0.05, $^{\#\#}$ p<0.01, $^{\#\#\#}$ p<0.001 for genotype comparison;

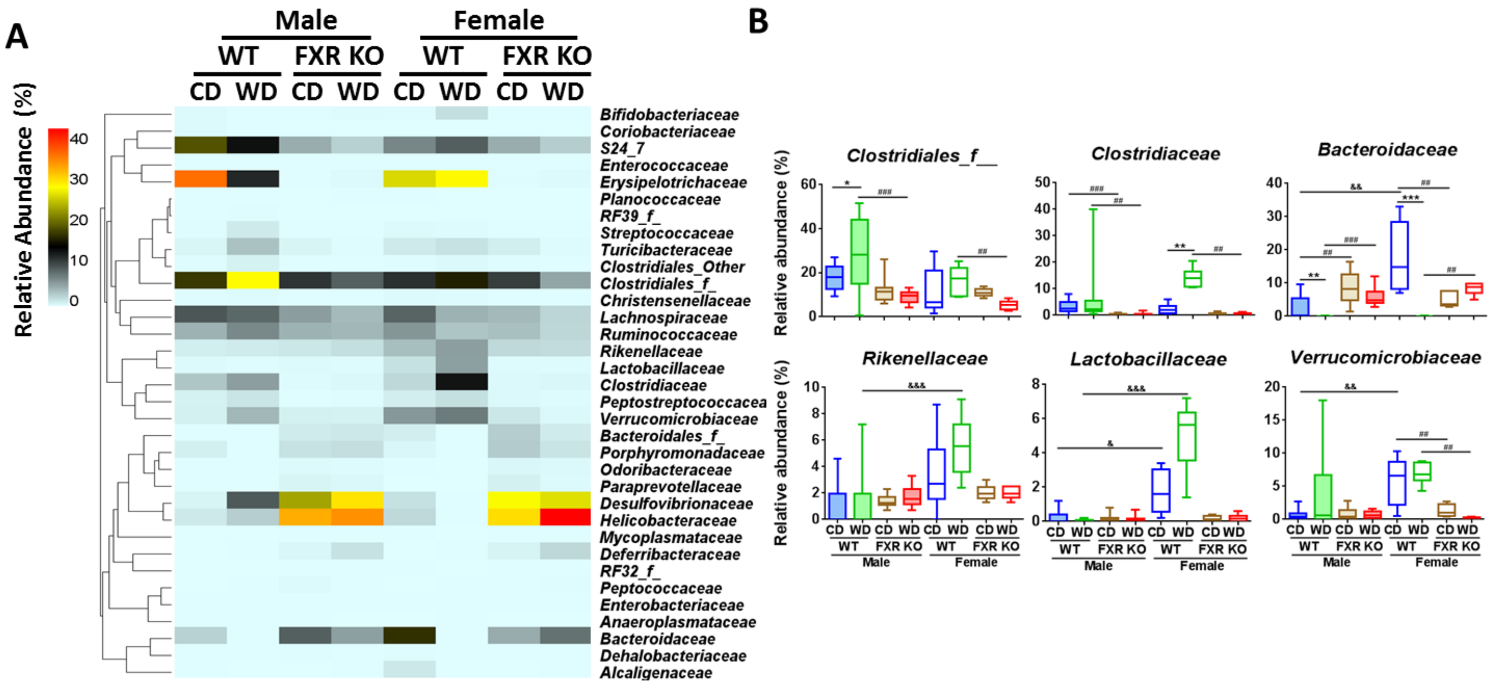

Fig. S3. Microbiota alteration based on diet, genotype, and gender at family level. (A) Relative abundance of cecal microbiota at family levels in control diet (CD) and Western diet (WD)-fed WT and FXR KO mice of both genders. Mean value was shown. (B) Relative abundance of cecal microbiota at family level in eight groups (Kruskal-Wallis test). Box plots display the median, 25th percentile, and 75th percentile; whiskers display minimum and maximum values. *n* = 16 in male groups, *n* = 6 in female groups. * *p*<0.05, ** *p*<0.01, *** *p*<0.001 for diet comparison; $^{\#}$ *p*<0.05, $^{\#\#}$ *p*<0.01, $^{\#\#\#}$ *p*<0.001 for genotype comparison; $^{\&}$ *p*<0.05, $^{\&\&}$ *p*<0.01, $^{\&\&\&}$ *p*<0.001 for gender comparison.

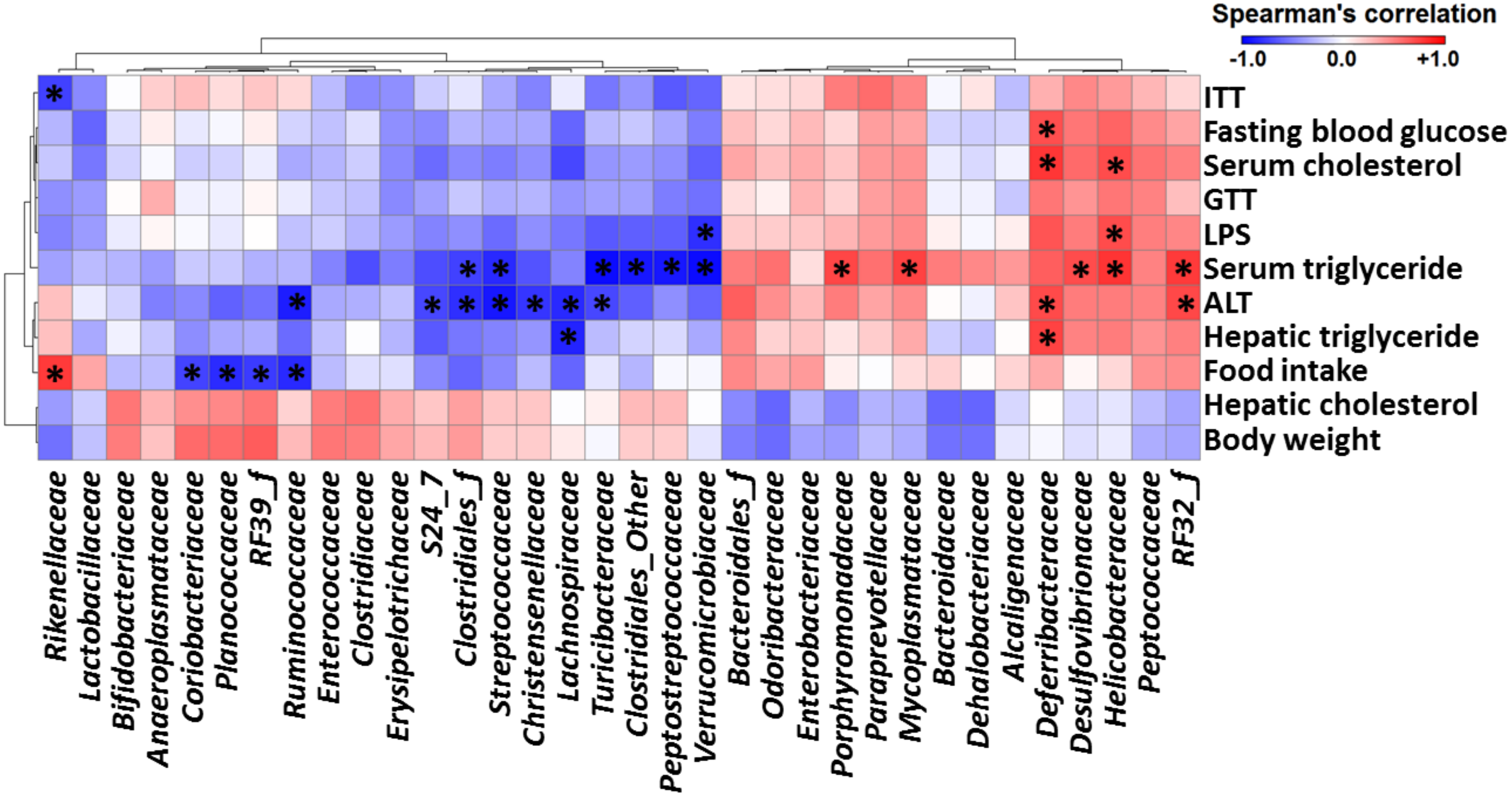

Fig. S4. Spearman's correlation analysis between the abundance of bacterial families and mouse phenotypes. * *p*<0.05.

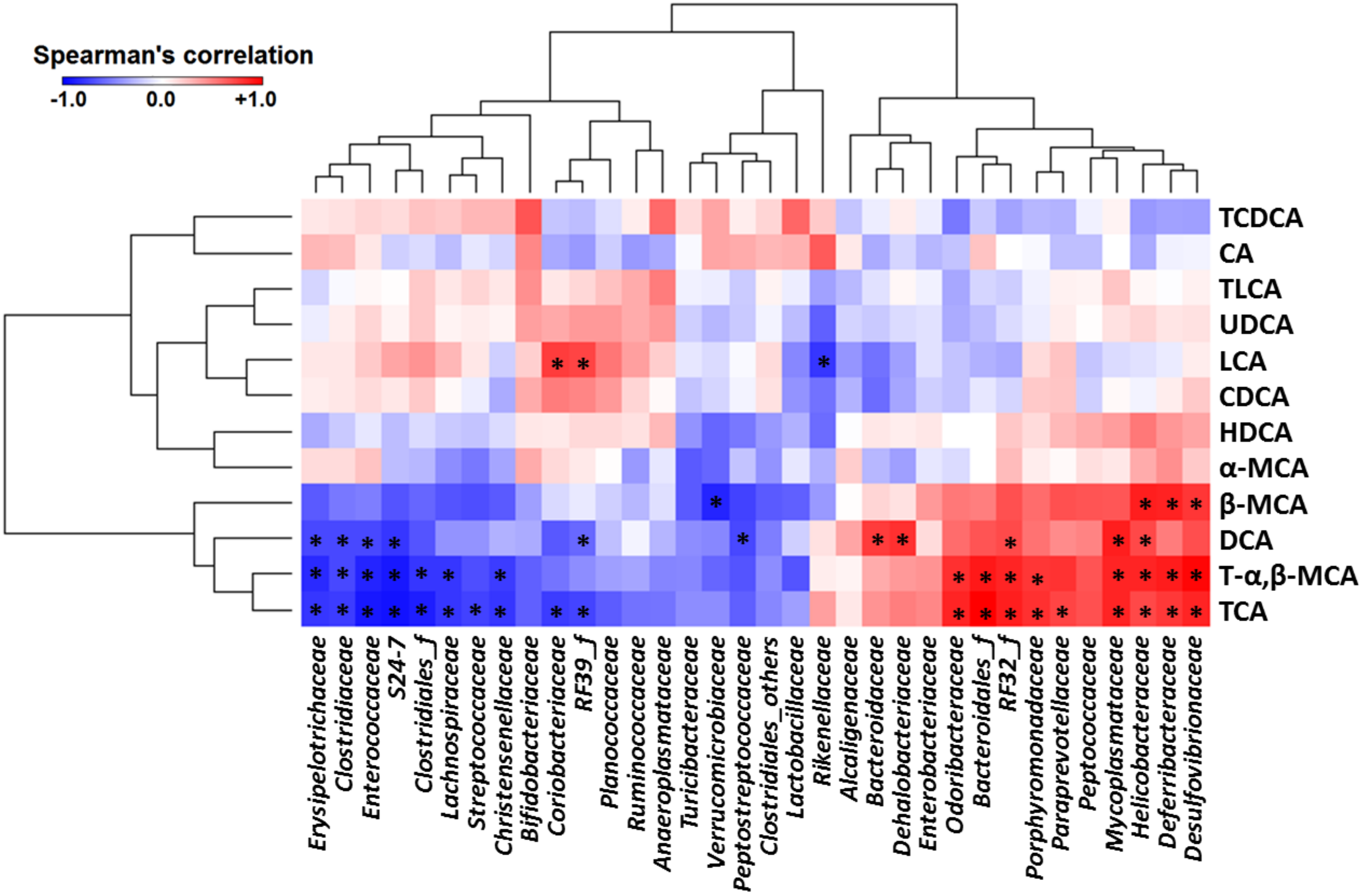

Fig. S5. Spearman's correlation analysis between the abundance of bacterial families and hepatic bile acids. * p<0.05.

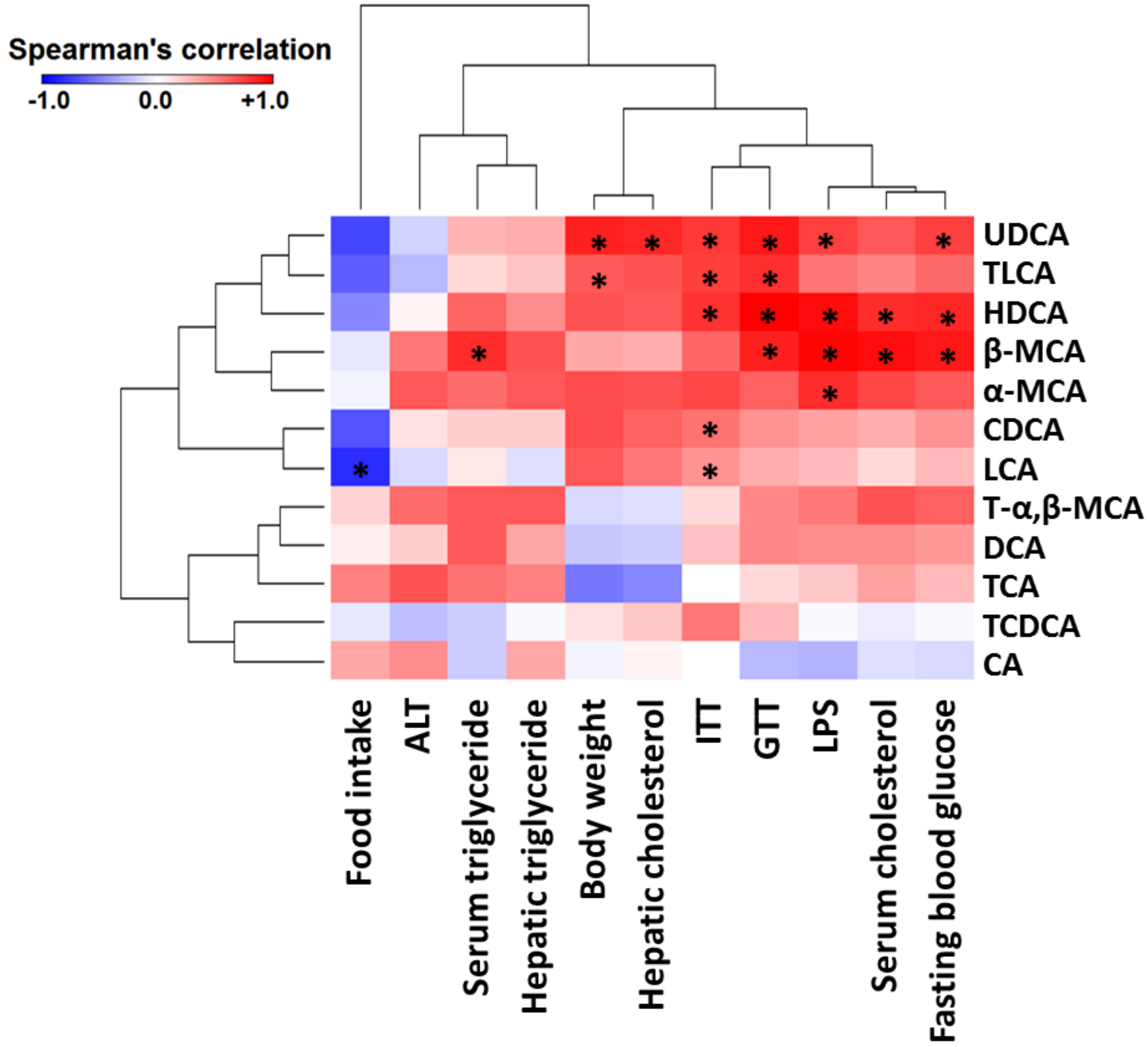

Fig. S6. Spearman's correlation analysis between mouse phenotypes and hepatic bile acids. * $p<0.05$.

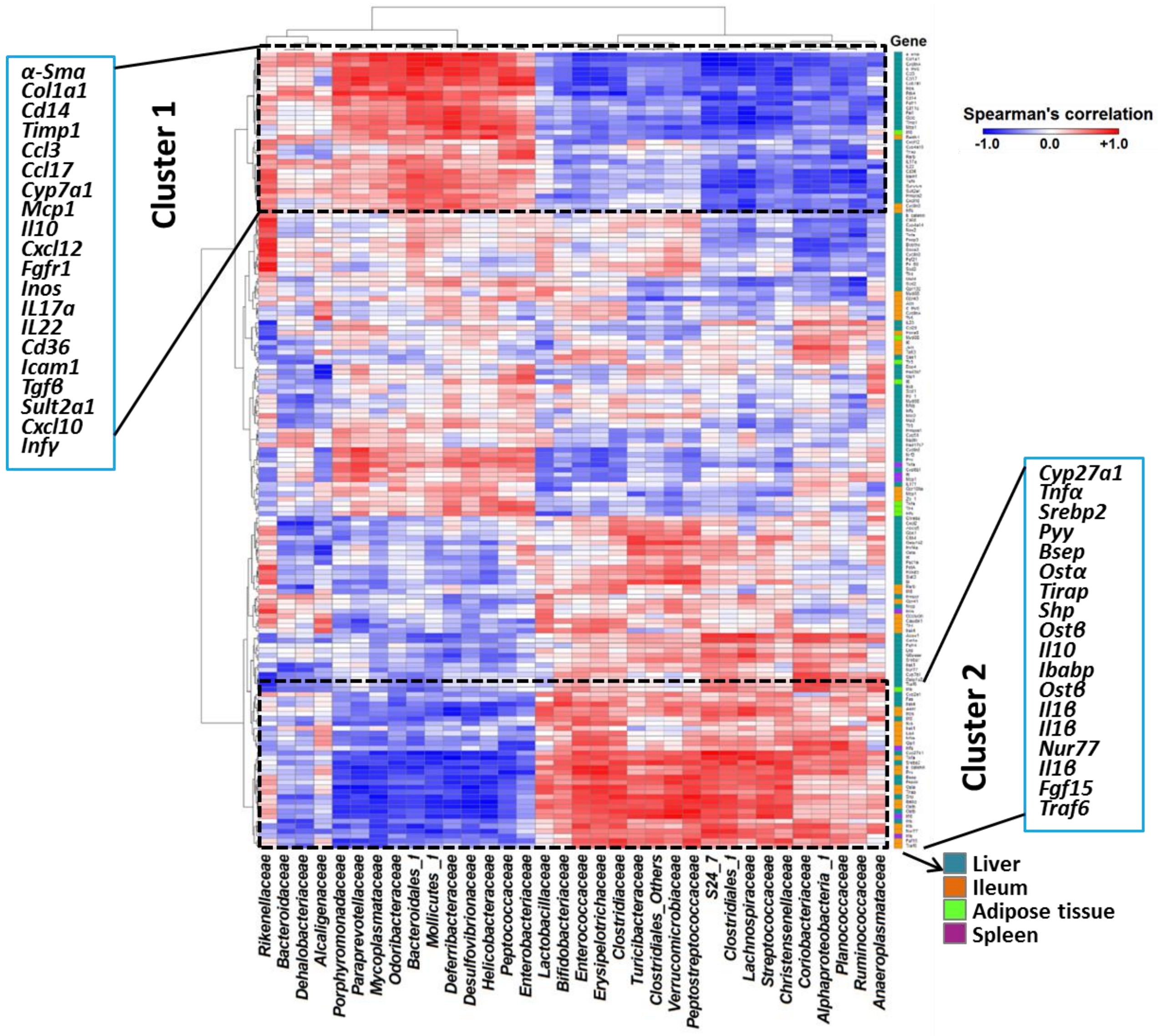

Fig. S7. Spearman's correlation analysis between the abundance of bacterial families and gene expression. * p<0.05.

REFERENCES

1 Yoshimoto, S. *et al.* Obesity-induced gut microbial metabolite promotes liver cancer through senescence secretome. *Nature* **499**, 97-101 (2014).

2 Louis, P., Flint, H.J. Development of a semiquantitative degenerate real-time PCR-based assay for estimation of numbers of butyryl-coenzyme A (CoA) CoA transferase genes in complex bacterial samples. *Appl Environ Microb* **73**, 2009-2012 (2007).

3 O'Keefe, S.J. *et al.* Fat, fibre and cancer risk in African Americans and rural Africans. *Nat Commun* **6**, 6342 (2015).

4 Ijssennagger, N. *et al.* Gut microbiota facilitates dietary heme-induced epithelial hyperproliferation by opening the mucus barrier in colon. *Proc Natl Acad Sci USA* **112**, 10038-10043 (2015).

5 Duary RK, Batish VK, Grover S. Relative gene expression of bile salt hydrolase and surface proteins in two putative indigenous Lactobacillus plantarum strains under in vitro gut conditions. *Mol Biol Rep* **39**, 2541-52 (2012).

6 Chandran A, Duary RK, Grover S*, et al.* Relative expression of bacterial and host specific genes associated with probiotic survival and viability in the mice gut fed with Lactobacillus plantarum Lp91. *Microbiol Res* **168**,555-62 (2013).
